# Reach of a Fully Digital Diabetes Prevention Program in Health Professional Shortage Areas

**DOI:** 10.1089/pop.2021.0283

**Published:** 2022-08-08

**Authors:** Lisa A. Auster-Gussman, Kimberly G. Lockwood, Sarah A. Graham, Natalie Stein, OraLee H. Branch

**Affiliations:** ^1^ Clinical Studies and Research, Lark Health, Mountain View, California, USA.; ^2^ Department of Psychiatry and Behavioral Sciences, University of California, San Francisco, California, USA.

**Keywords:** artificial intelligence, digital health, prediabetes, rural health, diabetes prevention program

## Abstract

The National Diabetes Prevention Program (NDPP) offers lifestyle change education to adults at risk for diabetes across the United States, but its reach is curbed due, in part, to limitations of traditional in-person programs. Diabetes Prevention Programs (DPPs) that are fully digital may increase reach by overcoming these barriers. The aim of this research was to examine the reach of Lark's DPP, a fully digital artificial-intelligence-powered DPP. This study assessed geographic features and demographic characteristics of a sample of Lark DPP commercial health plan members with complete data (*N* = 16,327) and compared several demographic features with a large composite sample of members from DPPs across the nation (NDPP; *N* = 143,489) and a National Health Interview Survey (NHIS) sample of prediabetic adults in the United States (NHIS; *N* = 2118). Examination of the Lark DPP sample revealed that 24.4% of members lived in rural areas, 30.8% lived in whole county health professional shortage areas, and only 7.6% of members lived in a zip code with an in-person DPP. When comparing the Lark sample with the NDPP and NHIS samples, Lark DPP enrollees tended to be younger and have a higher body mass index (BMI) (*p'*s < 0.001). Lark provides convenient access to a DPP for individuals living in hard-to-reach areas who may face barriers to participating in in-person or telephonic DPPs or who prefer a digital program. Compared with the NDPP sample, Lark is also reaching younger and higher BMI users, who are traditionally difficult to enroll and have a high need for intervention.

## Introduction

In the United States, 34 million adults have diabetes, and 88 million adults have prediabetes (13% and 34% of the population, respectively).^1^ Diabetes prevalence has increased in recent years, with corresponding increases in diabetes-related health complications.^1^ The National Diabetes Prevention Program (NDPP), recognized and monitored by the Centers for Disease Control and Prevention (CDC), has been working to reduce the incidence of type 2 diabetes (T2D) through healthy lifestyle education since 2010.^2^

Nearly 2000 CDC-recognized organizations provide the Diabetes Prevention Program (DPP) nationwide, primarily in-person.^3^ Since its inception, the DPP has seen great success, with >300,000 prediabetic individuals completing at least one DPP lesson from 2012 to 2019^4^ and significantly reducing participants' long-term risk of T2D.^5,6^ However, 300,000 is only a fraction of the millions of individuals living with prediabetes in the United States. As such, large-scale implementation of DPPs is an essential step toward reducing diabetes and prediabetes in the United States.

Recent estimates suggest that >100 million US adults, 43.5% of adults aged 20–64 years and 69.4% of adults ≥65 years old, are eligible for the DPP.^7^ Access to DPPs is increasing, but more rapid growth and alternate delivery approaches are needed.^4,8,9^ The vast majority of DPPs have been conducted in-person (76%) or through telehealth (11%), requiring in-person or phone counseling.^3^ Although these models are effective, they are labor and cost-intensive, making them difficult to scale.^10^ As a result, access to in-person DPPs is limited or unavailable for many underserved populations and/or individuals living in hard-to-reach areas, such as health professional shortage areas (HPSAs) or rural areas of the United States.

For example, only 14.6% of rural counties have a DPP site compared with 48.4% of urban counties.^11^ This lack of access is particularly concerning because rural areas and HPSAs typically have the highest rates of prediabetes and diabetes and the highest levels of socioeconomic deprivation.^12,13^ Given these disparities in DPP access and diabetes risk, it is crucial to develop scalable DPP solutions that increase access for all.

Digital platforms have the capacity to reach larger numbers of individuals, and to reach individuals who face any number of barriers to participating in traditional DPP programs.^8,10^ Commonly cited barriers center on the inconvenience of in-person programs, such as limited ability to travel to areas where programs are offered, limited time or time flexibility to participate in prescheduled lessons, and lack of childcare.^8,10,14^ These barriers may be particularly prevalent for individuals of lower socioeconomic status, who are also more likely to have prediabetes.^8^

Furthermore, prediabetic individuals with very high body mass index (BMI), who have the greatest likelihood of complications and comorbidities,^15^ and who pose the largest costs to medical systems and insurers^15,16^ are most likely to delay or avoid seeking health care and lifestyle change programs because of emotional barriers.^17^ A fully digital program powered by conversational artificial intelligence (AI) overcomes these barriers through on-demand access to the DPP in real time, when and where the individual needs it. In theory, these programs should increase access for those less likely to enroll in in-person DPPs, such as those living rurally or those with high BMIs. However, there is little published evidence on the demographics and geographic reach of digital DPPs.

Important next steps in this body of literature include describing the reach of a fully digital DPP and examining how digital DPP enrollees differ from both the historic enrollment in the DPP and from national statistics on adults with prediabetes in the United States. Herein, reach of the Lark DPP refers to characterization of individuals who downloaded the Lark application and completed at least one lesson.

Using data from Lark's DPP members, this study had two primary aims: (1) examined the reach of the Lark DPP based on demographic characteristics of a large sample of members (eg, age, sex, race, starting BMI, county socioeconomic status) as well as the geographic distribution of these individuals across Health and Human Services (HHS) regions, HPSA status, rurality designation, and traditional DPP access and (2) compared Lark members with broader samples of DPP enrollees and individuals with prediabetes in the United States.

## Materials and Methods

### The Lark diabetes prevention program

The Lark DPP is a 12-month digital program with full CDC recognition, the highest level of CDC certification (organization no. 4258176). Lark's AI-powered on-demand coaching is available 24 hours per day, 7 days per week. Members can access 26 integrated educational lessons that correspond to the Prevent T2 DPP curriculum.^18^

Lark coaches its members on health behaviors known to affect risk for T2D such as diet, physical activity, sleep, and stress, through integrated in-app tools. For example, the Lark program offers easy-to-use diet tracking powered by natural language processing. Physical activity tracking leverages both mobile phone motion sensors and connected fitness trackers, as well as manual entry. Members are encouraged to weigh-in using a cellular-connected digital scale provided by Lark as a part of the program. Because Lark coaching is powered by AI, it offers unlimited synchronous feedback, tips, and encouragement in real time.

#### Inclusion criteria

Lark members are primarily recruited through digital advertising on large platforms such as Facebook and e-mail campaigns executed by health plans or Lark. All Lark members have insurance plans that fully cover the Lark DPP. Members included in this study provided digital consent to use their de-identified data for research purposes. In addition, the analytic sample of 16,327 for the present analyses included only Lark members who opted to receive a digital scale and had complete data for basic demographic variables.

### Study design and measures

This study received exemption status from Advarra (Protocol no. Pro00047181) Institutional Review Board for retrospective analyses of previously collected and de-identified data. The demographic and geographic data from Lark members, as well as data and statistics from public repositories were used to examine how members of the Lark digital DPP platform compared on key demographic factors with a large sample of National DPP participants and to a representative US sample of adults with prediabetes.

#### Lark reach

Lark's reach was assessed by examining the demographic and geographic distributions of users who downloaded the Lark application and completed at least one lesson. Members entered their demographic data in-app, including their age, sex, height, and ethnicity, and starting BMI (kg/m^2^) was calculated using height and first weight. Average weight loss in the program was calculated based on the average of 14,342 members with available follow-up weight data after starting the program. The Lark DPP is provided through commercial health plans and does not require members to disclose socioeconomic information for program participation.

In lieu of individual-level socioeconomic data, zip code-level census data were used to provide general socioeconomic information for the Lark sample. Zip code-level socioeconomic variables (median household income, percent in zip code with different levels of education, and percent unemployment) were drawn from census data compiled by SimpleMaps (https://simplemaps.com/data/us-cities), downloaded on April 26, 2021. Complete data were not available for all zip codes, resulting in the following sample sizes: *N* = 13,193 (household income), *N* = 13,436 (education), and *N* = 12,808 (unemployment).

Lark's geographic reach was assessed using member geographic information (ie, zip code and county) that was matched to geographic data from several public repositories providing information on HHS region, rurality designation, HPSA status, and access to in-person DPPs. Members' HHS regions were determined using the regional map from HHS.gov (https://www.hhs.gov/about/agencies/iea/regional-offices/index.html) and rurality designations were determined using the Health Resources and Services Administration (HRSA)'s Federal Office of Rural Health Policy Health Policy definitions from this data set: https://www.hrsa.gov/rural-health/about-us/definition/index.html, downloaded on April 15, 2021.

To assess members' HPSA status, each county was categorized as a primary care whole, partial, or non-HPSA per the HRSA definitions using this data set: https://www.ruralhealthinfo.org/charts/5, downloaded on April 15, 2021. Finally, the percentage of Lark's members that had access to an in-person DPP within their zip code was examined. DPP access was determined from the National DPP registry: https://nccd.cdc.gov/DDT_DPRP/Registry.aspx on August 4, 2021.

#### Comparing the Lark DPP sample with the national DPP sample and US adults with prediabetes

To compare Lark demographics with participants enrolled in a broad sample of CDC-recognized DPPs, data published by the CDC Diabetes Prevention Recognition Program (DPRP) were utilized. Because DPPs are required to submit data biannually to the DPRP, this approach enabled us to compare the Lark sample with a broad sample of DPP participants. Summary statistics for the National DPP provided by Gruss and colleagues were used.^4^ This CDC article consolidated descriptive data from participants enrolled in DPPs across the United States from February 2012 to January 2019 who completed at least 3 lessons in the first 6 months. This article was selected for comparison because it provided the largest recent sample size published from DPRP data (NDPP; *N* = 143,489).

The data used to examine demographic characteristics of the US adults with prediabetes came from the 2019 National Health Interview Survey (NHIS; *N* = 31,997). The NHIS is designed to monitor the health of the US population and is one of the major data collection programs of the National Center for Health Statistics, which is part of the CDC. This data set was downloaded on August 15, 2021 from https://www.cdc.gov/nchs/nhis/2019nhis.htm and reduced by first removing all individuals who did not answer “Yes,” to the question, “Has a doctor or other health professional EVER told you that you had prediabetes or borderline diabetes?” Then, all members with any diabetes diagnosis (Type I, Type II, Gestational, and Don't know) were removed. This left a final sample of 2118 adults with prediabetes.

### Statistical analyses

All statistical analyses were conducted in RStudio 1.4.1106.^19^ For Aim 1, demographics and reach of the Lark sample were characterized using descriptive analyses. For Aim 2, the Lark sample demographics were compared with the NDPP and US adults with prediabetes using chi-square tests. These comparisons were limited to the variables with published summary statistics from the NDPP and corresponding variables available in the NHIS data set: age, sex, ethnicity, and starting BMI.

## Results

The first section of the results presented hereunder characterizes the Lark DPP sample. The second part of the results compares the Lark sample with the NDPP and NHIS samples on available variables.

### Lark demographic and geographic reach

The Lark analytic sample consisted of 16,327 Lark DPP members with complete baseline data. See Table 1 for means of continuous descriptive variables and Table 2 for sample proportions of categorical variables. Members ranged from 19 to 85 years old. As with most DPPs and weight loss programs, there were more females than males. The majority of the sample identified as white. Based on starting BMI, 98.6% of Lark DPP members were either overweight or obese, with a starting average weight of 104.1 kg (SE = 0.2 kg). Of those with available weight data (*N* = 14,324), the average weight loss was 4.1 kg (SE = 0.05 kg), with 81.8% of members losing weight while enrolled in the Lark DPP.

**Table 1.** Means for Member Sociodemographics

	Mean (SD) or* N *(%)
Age	46.3 (10.5)
Starting body mass index	37.0 (7.8)
Median household income in zip code^a^	
<$35,000	6139 (46.5%)
$35,000–49,999	3172 (24.0%)
$50,000–75,000	2160 (16.4%)
>$75,000	1722 (13.1%)
Education in zip code^a^	
% w/less than high school degree	14.8 (13.7)
% w/high school degree	30.6 (12.0)
% w/some college	29.4 (9.0)
% w/bachelor's degree	17.1 (10.9)
% w/graduate degree	10.4 (9.4)
Unemployment rate in zip code^a^	5.4% (3.7)

^a^ Denotes variables derived from zip code-level census data. Sample sizes: age and body mass index, *N* = 16,327; household income, *N* = 13,193; education, *N* = 13,436; unemployment, *N* = 12,808.

**Table 2.** Comparisons of Member Sociodemographics and Characteristics

	Lark DPP sample (*N* = 16,327)	NDPP sample (*N* = 143,489)	NHIS prediabetic sample (*N* = 2118)
Age (years)			
18–44	41.0%	30.6%	17.1%
45–64	55.3%	54.5%	39.3%
65+	3.8%	14.9%	43.5%
		χ^2^ = 20343.0^**^	χ^2^ = 3919.7^**^
Starting body mass index			
Overweight	16.6%	27.3%	31.7%
Obese	81.9%	72.3%	40.0%
		χ^2^ = 821.1^**^	χ^2^ = 505.88^**^
Sex			
Female	77.1%	75.3%	54.3%
Male	22.9%	24.7%	45.7%
		χ^2^ = 25.7^**^	χ^2^ = 513.58^**^
Race			
White (non-Hispanic)	73.4%	64.6%	65.5%
Black (non-Hispanic)	10.6%	11.9%	12.2%
Hispanic or Latino	10.0%	9.0%	13.6%
Other (non-Hispanic)	6.0%	14.5%	8.6%
		χ^2^ = 987.7^**^	χ^2^ = 63.99^**^

χ^2^values indicate tests comparing the Lark sample with the NDPP sample and to the NHIS prediabetic sample in separate analyses.

^**^ <0.01.

DPP, Diabetes Prevention Program; NDPP, National Diabetes Prevention Program; NHIS, National Health Interview Survey.

The mean of the median household income for Lark DPP members' zip codes was $38,433, and nearly half the sample lived in areas where median household income was <$35,000. Lark members lived in zip codes where 15% of residents had less than a high school education, 31% had a high school degree, 29% had completed some college, 17% of people had a bachelor's degree, and 10% had a graduate degree. Average unemployment rate across members' zip codes was ∼5%.

#### HHS region

**FIG. 1.**
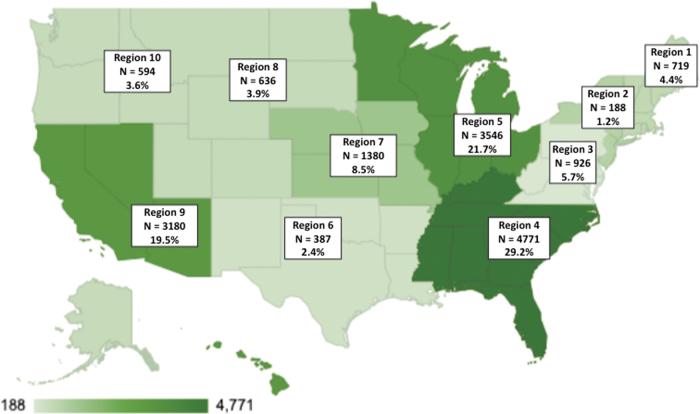
Lark members by HHS region. HHS, Health and Human Services.

Lark DPP members resided in all 10 US HHS regions, with the largest proportion drawn from HHS regions 4 (29.22%, *N* = 4771), 5 (21.72%, *N* = 3546), and 9 (19.48%, *N* = 3180). Figure 1 shows the distribution of Lark members by HHS region.

#### HPSA designation

**FIG. 2.**
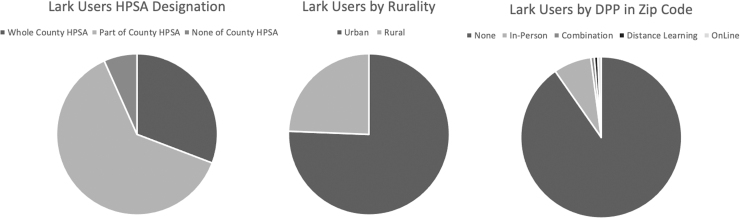
Lark members by HPSA designation, rurality, and DPP access type. DPP, Diabetes Prevention Program; HPSA, health professional shortage area.

The majority of Lark DPP members lived in counties that were partially or wholly classified as primary care HPSAs. Specifically, 30.78% (*N* = 5026) lived in an area designated as a whole county HPSA and 62.53% (*N* = 10,209) lived in a partial county HPSA. Only 6.68% (*N* = 1092) of members lived in a non-HPSA county (Fig. 2).

#### Rurality designation

Based on HRSA designations, 24.37% (*N* = 3979) of Lark DPP members resided in a rural area and 75.63% (*N* = 12,348) lived in an urban area (Fig. 2).

#### Access to in-person DPPs

Most Lark DPP members did not have an in-person DPP in their zip code: only 7.61% (*N* = 1242) of Lark DPP members had an in-person DPP in their zip code, whereas 90.32% did not have any form of in-person DPP in their zip code (Fig. 2).

### Demographic comparisons

#### Age groups

There were age distribution differences between the Lark members and the NDPP sample and differences between Lark and the NHIS sample (Table 2). Lark enrolled a larger proportion of younger members (ie, 18–44) compared with both the NDPP and the NHIS sample.

#### Starting BMI

**FIG. 3.**
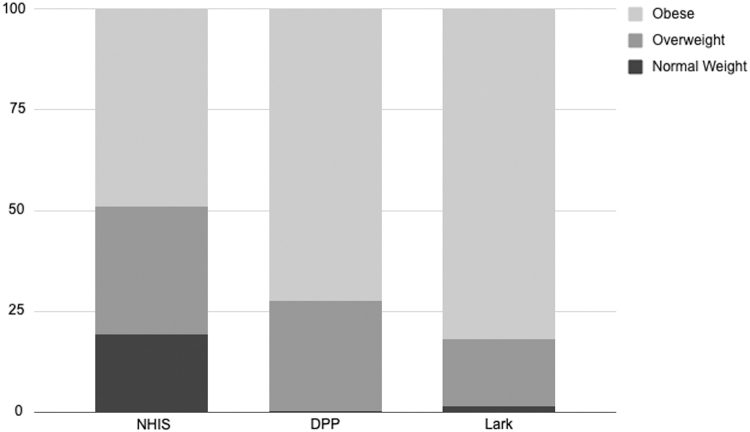
Proportion of individuals in each BMI category for NHIS, NDPP, and lark samples. BMI, body mass index; NDPP, National Diabetes Prevention Program; NHIS, National Health Interview Survey.

The percentage of obese individuals enrolled in the Lark DPP was significantly higher than both NDPP and the NHIS samples (Table 2). Figure 3 shows the proportion of individuals classified as normal weight, overweight, and obese for the NHIS, NDPP, and Lark samples. The higher baseline BMI for Lark was not explained by having different inclusion criteria because Lark uses the same starting BMI of ≥25.

#### Sex

Compared with both the NDPP and NHIS samples, Lark had a higher proportion of females compared with males, but the significance level was likely influenced by the large sample size and unlikely to be meaningfully different between the Lark sample and NDPP sample with a difference of only 1.8% (Table 2).

#### Race/ethnicity

The Lark DPP sample differed from both the NDPP and NHIS samples in terms of the distribution of race/ethnicity (Table 2). This overall difference appeared to be driven primarily by differences in the percentage of white non-Hispanic individuals.

## Discussion

### Main findings

This study described the reach of the Lark DPP for active participants who completed at least ≥1 lesson of the program and compared the Lark DPP sample with participants in the broader NDPP and a national sample of adults with prediabetes in the United States. The geographic analyses indicated that a large proportion of Lark members were located in areas with limited access to health care, such as rural regions, whole or partial HPSAs, and zip codes with no CDC-recognized in-person DPP. In addition, the demographic comparisons indicated that the Lark DPP enrolled members were younger and of higher BMI than participants in the broader NDPP and typical adults with prediabetes in the United States.

The geographic analyses demonstrated the reach of the Lark DPP. We found that nearly 50% of Lark members were located in HHS regions 4 and 5, which include the majority of the Southern states and several Midwestern states. Notably, HHS regions 4 and 5 include most of the “Stroke Belt” states and comprise 13 of the 20 states with the highest rates of diabetes in the United States,^20^ indicating that Lark reached areas with particularly high diabetes risk. Lark member data also showed that 24% of the sample resided in rural areas. This value indicates that rural participants were overrepresented in the Lark sample compared with the broader US population, of which ∼20% live in rural regions.^21^ Rural areas also tend to have the highest rates of diabetes risk^12,13^ and are less likely to have access to an in-person DPP compared with urban areas.^11^

In addition, >90% of the Lark sample did not have access to an in-person DPP in their zip code, indicating that lack of convenient physical proximity to DPPs may prevent many Lark members from participating in an in-person lifestyle intervention. Given that physical location and convenience are well-documented barriers to participating in a DPP,^8,10,14^ Lark may be providing prediabetic individuals with a convenient, on-demand, and much-needed solution to reducing their diabetes risk.

These analyses also showed that 93.3% of Lark members were located in either partial (62.5%) or whole county (30.8%) HPSAs, demonstrating that the Lark DPP reached individuals who may have significant issues accessing primary care providers and/or facilities. Notably, this 93.3% exceeds national statistics showing that 89% of US counties are whole or partial county HPSAs.^22^ In summary, the geographical analyses indicated that Lark is enrolling individuals in high-risk areas of the United States who may be unable to access primary care or lifestyle interventions that are crucial for treating prediabetes.

The demographic comparisons showed that Lark had a higher proportion of younger members compared with the NDPP and NHIS samples. This finding is particularly interesting because past research has noted that the DPP has struggled to enroll younger individuals,^1,10^ and younger adults are typically less engaged in chronic disease prevention programs such as the DPP.^23^ In addition, the US Preventive Services Task Force recently recommended lowering the prediabetes and diabetes screening age to 35 years, further highlighting the need to reach younger prediabetic individuals.^24^ It is likely that Lark's recruitment, which uses a number of online mechanisms such as social media, rather than in-person materials, may have contributed to younger members finding and choosing to engage with the Lark DPP.

This is supported by literature showing that social media platforms are effective at reaching a younger audience for diabetes management program recruitment.^25^ In addition, the Lark sample likely included more younger adults because digital DPPs are not currently a covered benefit for Medicare and Medicare Advantage members ≥65 years. That said, prior research has shown that Lark members >65 years have the highest engagement among all users.^26^ Regardless, these data provide encouraging evidence that younger members will enroll in digital DPPs at higher rates than typically observed in the DPP nationally.

Overall, Lark had more members with a BMI classified as obese compared with the NDPP sample and compared with the NHIS sample. Individuals with the highest BMI have the highest risk for progression from prediabetes to T2D, as well as the greatest potential for future disease-related complications.^15,16^ Research also suggests that individuals with very high BMI are more likely to avoid health care visits and health-related programming because of perceived weight stigma.^17^ These findings suggest that some of these stigma-related concerns may have been avoided due to Lark's AI-powered fully digital interaction.

Across many DPPs, there is an opportunity to increase enrollment among non-white individuals. For example, results from another large-sample DPP study that, similar to Lark, used population health management strategies to recruit, showed that participants were more likely to be white than census tract data would predict.^27^ This suggests that many DPPs, including digital programs, must work to increase engagement among minoritized racial groups in the United States.

### Strengths and limitations

The primary strength of this study was the large sample size, allowing for a comprehensive picture of individuals who engaged with the Lark DPP. This article is among the first to offer these types of insights into such a large number of members of a fully digital DPP. This study contributed to the body of knowledge regarding how individuals who enroll in digital DPPs differ from the typical NDPP population and prediabetic individuals in the United States.

However, the study had several limitations. The data did not allow for direct comparison of the geographic reach outcomes (such as % in HPSAs) among Lark, the NDPP, and the NHIS samples because of a lack of access to data on NDPP samples' county or zip codes. Reach also only included individuals who were active in the DPP programs. An analysis of those who encounter marketing of the program regardless of whether they engage with the program would be a valuable question for future research. In addition, the socioeconomic data used to characterize Lark members were based on zip code census data, rather than direct report from participating individuals; this is an artifact of using a real-world functioning app for which the enrollment process did not require reporting of these sensitive variables.

The state locations (such as what is used by HHS regions) are inherently impacted by Lark's insurance partners' locations because Lark is offered solely as an insurance benefit for covered members. The sample, by the nature of how Lark is accessed, is also limited in generalizability because all users gain access to Lark through commercial insurance providers. Finally, given that Lark aims to serve individuals in rural areas, there may be connectivity issues as a result of insufficient broadband or cellular data access for full app functionality in some rural locations.

## Conclusions

This article highlights the broad reach of a fully digital DPP across the United States. This study provided important insights into the ways in which fully digital DPPs compare with, and may extend, in-person DPP offerings. In particular, these findings showed how Lark increased access to DPPs for individuals living in hard-to-reach areas (eg, rural regions, HPSAs) and for those who did not have convenient access to an in-person DPP. The results of this research further demonstrated that a fully digital solution may be particularly beneficial for younger adults and/or individuals with a high BMI. Unlike in-person DPPs, the opportunity for a fully digital program to provide DPP access to more people is boundless, as the AI is infinitely available and scalable.
